# Characterisation of Commensal *Escherichia coli* Isolated from Apparently Healthy Cattle and Their Attendants in Tanzania

**DOI:** 10.1371/journal.pone.0168160

**Published:** 2016-12-15

**Authors:** Balichene P. Madoshi, Egle Kudirkiene, Madundo M. A. Mtambo, Amandus P. Muhairwa, Athumani M. Lupindu, John E. Olsen

**Affiliations:** 1 Department of Veterinary Medicine and Public Health, Sokoine University of Agriculture, Morogoro, Tanzania; 2 Livestock Training Agency – Mpwapwa Campus, Mpwapwa, Dodoma; 3 Department of Veterinary Disease Biology, Faculty of Health and Medical Sciences, University of Copenhagen, Frederiksberg, Denmark; 4 Tanzania Industrial Research Developments Organisation, TIRDO Complex, Dar es Salaam, Tanzania; Seconda Universita degli Studi di Napoli, ITALY

## Abstract

While pathogenic types of *Escherichia coli* are well characterized, relatively little is known about the commensal *E*. *coli* flora. In the current study, antimicrobial resistance in commensal *E*. *coli* and distribution of ERIC-PCR genotypes among isolates of such bacteria from cattle and cattle attendants on cattle farms in Tanzania were investigated. Seventeen *E*. *coli* genomes representing different ERIC-PCR types of commensal *E*. *coli* were sequenced in order to determine their possible importance as a reservoir for both antimicrobial resistance genes and virulence factors. Both human and cattle isolates were highly resistant to tetracycline (40.8% and 33.1%), sulphamethazole-trimethoprim (49.0% and 8.8%) and ampicillin (44.9% and 21.3%). However, higher proportion of resistant *E*. *coli* and higher frequency of resistance to more than two antimicrobials was found in isolates from cattle attendants than isolates from cattle. Sixteen out of 66 ERIC-PCR genotypes were shared between the two hosts, and among these ones, seven types contained isolates from cattle and cattle attendants from the same farm, suggesting transfer of strains between hosts. Genome-wide analysis showed that the majority of the sequenced cattle isolates were assigned to phylogroups B1, while human isolates represented phylogroups A, C, D and E. In general, *in silico* resistome and virulence factor identification did not reveal differences between hosts or phylogroups, except for *lpfA* and *iss* found to be cattle and B1 phylogroup specific. The most frequent plasmids replicon genes found in strains from both hosts were of *IncF* type, which are commonly associated with carriage of antimicrobial and virulence genes. Commensal *E*. *coli* from cattle and attendants were found to share same genotypes and to carry antimicrobial resistance and virulence genes associated with both intra and extraintestinal *E*. *coli* pathotypes.

## Introduction

*Escherichia coli* is an abundant commensal bacterium in the gastrointestinal tract of all warm-blooded animals. Recent epidemiological and genome-wide sequence analysis suggest that there is no clear line between the commensal and pathogenic *E*. *coli;* as a group they share most of the pathogenicity factors and belong to the same pathotypes and phylogroups, and are host independent [1, 2]. It becomes evident that the pathogenicity of *E*. *coli* is dependent on the regulation and interaction between a number of virulence factors, and it is effected by environmental conditions e.g. host species, host health status, interaction with other bacteria species *etc*. Due to this, under certain, yet unknown circumstances, any *E*. *coli* isolate carrying pathogenicity or antimicrobial resistance genes is potentially pathogenic and harmful to its host.

Cattle regularly excrete pathogenic [3] and non-pathogenic *E*. *coli* [4]. Cattle-related intestinal *E*. *coli* isolates have been reported to be transmissible to human [5] leading to concern of human health, more so in developing countries where there is rapidly expanding peri-urban population and, concurrent with this, cattle populations in the same areas, combined with lack of infrastructures to handle animal wastes appropriately. In addition, antimicrobial resistance genes transmission has been perpetuated in many bacterial populations due to use of antimicrobials in animal feeds [6–8] as well as irrational use of antibiotics in humans and cattle [9]. Contrary to the pathogenic subtypes, little is known about commensal *E*. *coli* from cattle, and the risk that they may transfer resistance plasmids, resistance genes and virulence factors to isolates of *E*. *coli* residing in the human intestine.

Correlation in the increase of antimicrobial resistance of *E*. *coli* in animals and farm workers has been documented [10–12], yet the direct transfer of genetic material between the hosts was not proved, as common infection source for different hosts could exist. Nevertheless, the presence of the same *E*. *coli* genotypes in cattle, poultry and farm/slaughterhouse workers as well as share of specific plasmids, carrying virulence and antimicrobial resistance genes, indicate that genome content can be directly transferred between *E*. *coli* colonizing different hosts and increase the risk of *E*. *coli* infections to a general population *per se*. In this study the antimicrobial resistance and genetic relatedness of *E*. *coli* populations from apparently healthy cattle and their attendants in cattle farms in Tanzania was analyzed. To investigate the pathogenic potential of the commensal *E*. *coli* and possible exchange of the genomic content between cattle and human isolates, we further compared selected genomes of *E*. *coli* from both hosts.

## Materials and Methods

### Study area, sample collection and isolation of *E*. *coli*.

The study was carried out in 13 wards of Morogoro Urban and Peri-urban areas, Tanzania, where faecal samples of cattle from 137 cattle herds and 50 cattle attendants from these herds were obtained. Selection of herds was based on lists of cattle herders kept by village authorities and a randomized selection procedure, however, the selection was not totally random, as consent from herd owners was needed. Approximately ten grams of faecal sample were collected per rectal using gloved hand from clinically healthy cattle, while attendants, under their consent, were given sterile containers to provide stool. The National Institute for Medical Research (NIMR) approved the study to be conducted in human subjects (permit number NIMR/HQ/R.8a/Vol.IX/1883). In preparation for the sampling from cattle, the animal care and use committee of Sokoine University of Agriculture, Tanzania, issued a permit (SUA/FVM/R.1/9 of 28th March 2014) with recommendation of using a professional Veterinarian to collect the samples. Philbert Balichene, who is a registered Veterinarian in Tanzania and 1st author of the manuscript, collected the samples. The samples were placed in a cool box, transported to the laboratory and processed on the same day. A gram of the faecal sample was diluted in 9 ml of sterile normal saline and 100 μl of the aliquot was spread on MacConkey Agar (Oxoid Ltd, Hampshire, England) using a sterile loop wire and incubated at 37°C for 24 hours. One pinkish-red dry colony with a diameter >0.5 mm per sample was randomly picked and subcultured once more on MacConkey agar under same conditions. Purified strains were further identified using Gram-stain and biochemical tests (Indole, Methyl red, Voges Proskaeur and Citrate utilization) and stored in 30% sterile glycerol at -80°C for further analysis.

### *E*. *coli* species confirmation

Preserved isolates were sub-cultured on Brain heart infusion agar with 5% calf blood for 24hrs at 37°C. A small amount of the individual colonies of the fresh bacterial cultures were transferred to a MALDI-MS target plate using a sterile pipette tip. Immediately after deposition samples were overlaid with 1.0 μl of a CHCA matrix solution (Vitek^®^ MS-CHCA; bioMérieux SA) and allowed to dry at room temperature. Spectra were acquired from samples on a VITEK^®^ MS RUO instrument (bioMérieux, Marcy l'Etoile, France) in linear, positive ion extraction mode in a mass range from 2 to 20 kDa, with a laser frequency of 50Hz, an acceleration voltage of 20 kV, and an extraction delay time of 200 ns. Spectra were acquired in an automatic mode, by accumulating 100 profiles of 5 laser shot cycles each using the auto-quality control of Launchpad 2.9. The instrument was calibrated by using an *Escherichia coli* reference strain (ATCC 8739), which was prepared according to the manufacturers specifications and transferred to the designated wells on each target slide. Isolates which were identified with a low confidence (<85%) in MALDI-TOF were sub-cultured on chromogenic *E*. *coli* rapid agar at 37°C for 24 hour as described by Abulreesh [13]. Positive isolates, which formed large bluish purple colonies in the media, were included in the study, while isolates with different colours were disregarded.

### Enterobacterial Intragenic Consensus—Polymerase Chain Reaction (ERIC—PCR) fingerprinting

Following MALDI-TOF confirmation, one *E*. *coli* colony per strain was suspended in 100μl sterile miliQ water in eppendorf tubes, boiled for 10 minute at 95°C without shaking and centrifuged for 5 minute at 13.000 rpm. The resulting supernatant was pippetted (80μl) and stored at -20°C for further analysis. DNA quantity and quality were assessed using spectrophotometer Nanodrop 1000 (Thermo Fisher Scientific, U.S.A). Each *E*. *coli* isolate was analyzed using ERIC-PCR primers [14] and conditions previously described [15]. A negative control (sterile milliQ water) was included in every PCR reaction, while *E*. *coli* K-12 (ATCC 25922 strain) was included as positive control to assess the reproducibility of the study. The PCR products were electrophoresed in 1.5% agarose gels stained with ethidium bromide at 100 V for 45 minutes and 75 V for 10 minutes, and band patterns were captured under UV illuminator. GeneRuler 1-kb plus molecular weight marker (Thermo Fisher Scientific, U.S.A) was loaded as a standardized reference. GelCompar 4.6 software (Applied Maths, Belgium) was used to compare the gels and to generate the phylogenetic tree based on Pearson correlation coefficient and Unweighted paired group mean and Arithmetics (UPGMA) method of clustering.

### Antimicrobial sensitivity Test

Disk diffusion test was carried as described by Bauer et al. [16] as per EUCAST (2015) guidelines. From three to four colonies were dissolved in 10 ml of 0.9% saline. The density was measured on a pre-calibrated nephelometre (Sensititre Nephelometer Thermo Scientific, Denmark) to 0.5 McFarland turbidity. The antimicrobial discs used in this study were from Oxoid Ltd., (England) and included: Ciprofloxacin (CIP 5μg), Chloramphenical (C 30μg), Gentamicin (CN 10μg), Cefotaxime (CTX 5μg), Cefoxitin (FOX 30μg), Colistin Sulphate (CT 10μg), Ampicillin (AMP 10μg), Amoxicillin—Clavulanic Acid (AMC 30μg/), Sulphamethoxazole—Trimethoprim (SXT 19:1) and Tetracyclines (TE 30μg). *E coli* ATCC 25922 was used as an internal positive control. Differences in resistance prevalence between *E*. *coli* isolated from human and cattle were evaluated using Pearson chi-square test.

### Sequencing of *E*. *coli* genomes

Seventeen *E*. *coli* isolates were selected to represent different geographical location, ERIC-PCR groups and the source of isolation. The isolates were grown in Luria broth for 16 h and genomic DNA was isolated using blood and tissue kit (catalog no. 69506; Qiagen) according to the instructions of the supplier. Genome sequencing was performed using the MiSeq instrument (Illumina) at a 300-bp paired-end-read format. Sequencing reads were *de novo* assembled using the SPAdes v. 3.5.0 [17]. The genome sequences from the 17 *E*. *coli* strains were submitted to Genbank (BioProject ID: 293513). Detailed genome sequences statistics and accession numbers are provided in S1 Table.

### *In silico* analysis of genome sequences

Multilocus Sequence Typing (MLST) tool [18] was used to identify the sequence type (ST type) from the assembled *E*. *coli* genomes. The tool reports the best match in case the alleles do not show a perfect match with the known alleles. In such cases, new alleles and ST types were obtained by performing PCR amplification as described previously [19]. Novel alleles were deposited to the public *E*. *coli* MLST database (http://mlst.warwick.ac.uk/mlst/mlst/dbs/Ecoli).

The Centre for Biological Sequence analysis (CBS) servers: PlasmidFinder [20], SeroTypeFinder, VirulenceFinder [21] and ResFinder [22] were employed for the *in silico* prediction of plasmid associated replicons, the serotype of the strains, genes associated with *E*. *coli* virulence and antibiotic resistance genes. The threshold for reporting a match between a gene in the PlasmidFinder and SeroTypeFinder databases and the input genome was set to be 80% identity across at least 60% of the length of the gene in the databases. For a hit to be reported by VirulenceFinder and ResFinder, it had to cover at least 60% of the length of the gene sequence in both databases with the sequence identity of 85% and 60%, respectively. Phylogroups of *E*. *coli* genomes were determined using phylotyping primers for the *E*. *coli* phylogroups A, B1, B2, C, D, E, F [23, 24].

### Evolutionary relationship analysis

The relationship of the strains was inferred using seven housekeeping locus fragments (*adk*, *fumC*, *gyrB*, *icd*, *mdh*, *purA* and *recA*) retrieved from the sequenced genomes. The corresponding genes of each strain were aligned with MAFFT v7.130b [25], and concatenated using *catfasta2phyml*.*pl* script. Further the Maximum Likelihood method based on the Hasegawa-Kishino-Yano model implemented in the MEGA v.6 [26] was used to create a phylogenetic tree. The core genome Single nucleotide polymorphisms (SNPs) tree were constructed using Conserved Signature Indels (CSItree) available through the Centre for Biological Sequence (CBS) [27] where *E*. *coli* K12 strain MG1655 (accession no. U00096) was used as a reference genome.

### Host and lineage specific gene detection

GET_HOMOLOGUES software [28] was employed to calculate the intersect-pangenome of the 17 *E*. *coli* strains, which was used to identify the presence/absence of genes between the different sample sets (host or ERIC groups). The genes were considered sample set specific when they were present in 90% genomes of one sample set and absent in 90% genomes of the other sample set.

## Results

### Isolate confirmation and antimicrobial resistance

This study involved 137 *E*. *coli* isolates obtained from cattle and 50 isolates from attendants of these animals (Table 1).

**Table 1:** The number of *E*. *coli* isolates per location.

Ward	Ward Identity	Cattle	Human
Bigwa	a	6	2
Boma	b	14	2
Kichangani	c	5	3
Kihonda	d	1	1
Kihonda Magorofani	e	18	15
Kingorwila	f	11	5
Mafisa	g	13	12
Magadu	h	21	5
Mazimbu	i	42	5
Mkundi	j	1	0
Tungi	k	5	0
Total		137	50

The isolates were confirmed by MALDI–Tof and Brilliance *E coli* Agar to belong to the species *E*. *coli*. All confirmed isolates were tested for the resistance to ten antibiotics. The analysis revealed that tetracycline (TE), sulphamethazole-trimethoprim (SXT) and ampicillin (AMP) were the most frequent resistance types both among human and cattle isolates (Table 2). Human isolates were significantly more resistant to STX and AMP compared to cattle, and additionally showed high resistance of 20.4% towards cefotaxime (CTX). Resistance to only one of the tested antimicrobial was frequently found among cattle isolates (34.1%) and was less frequent in human ones (6.4%). Both human and cattle isolates shared similar levels (10.6% and 9.4%) of resistance to two different antimicrobial classes, where the most common antimicrobial combinations were either TE/AMP or TE/SXT. Resistance to more than two antimicrobial classes (multi-resistance) was more frequent among human isolates than among the cattle (49.0% versus 10.7%).

**Table 2:** Antimicrobial resistance of 185 *E*. *coli* isolates from cattle and cattle attendants in Tanzania.

Antibiotic type(s)	Cattle, % (n = 137)	Human, % (n = 50)	*X*^*2*^	*p-value*^a^
Tetracycline (TE)	33.1	40.8	0.6	0.38
Gentamycin (CN)	2.9	6.1	0.3	0.38
Sulphamethazole—Trimethoprim (SXT)	8.8	49.0	34.5	**<0.001**
Ampicillin (AMP)	21.3	44.9	8.5	**0.002**
Chloramphenical (C)	4.4	6.1	0.7	**<0.001**
Ciproflaxacin (CIP)	0.7	4.1	0.9	0.17
Cefotaxime (CTX)	3.7	20.4	11.4	**<0.001**
Colistin Sulphate (CT)	1.5	2.0	0.2	1.00
Ampicillin—Clavulanic (AMC)	5.2	10.2	0.8	0.31
Cefoxitin (FOX)	0.0	0.0	0.6	0.38

^a^ p-value for difference between prevalence among *E*. *coli* from humans and cattle.

### Genetic diversity of *E*. *coli* isolates

Out of 187 isolates subjected to antimicrobial resistance testing, ERIC-PCR type was obtained from 172 isolates. In total 66 genotypes were revealed when a similarity cut-off of 90% was used. Out of these types, 14 and 36 types were found to be unique to human and cattle samples, respectively, while 16 types were shared between the two hosts. Among the shared genotypes, seven (Types 19, 34, 45, 48, 49, 56, 57; see Table 3) contained isolates from human and cattle from the same ward, indicating that strains were shared between the two hosts. Genotype 25 representing 17 isolates from both human and cattle in eight wards was the most common genotype. Other common genotypes, represented by 6–12 isolates from several wards and both hosts, were genotypes 49, 11, 51, 8,12, 27, 56, 58 and 19. The remaining genotypes were found in <6 isolates each. In total, 32 genotypes were ward specific, and 19 genotypes shared between different wards (Table 3).

**Table 3:** Distribution of ERIC-PCR genotypes among 172 *E*. *coli* isolated from human and cattle in in Tanzania.

Ward ID	No of genotypes/(no of isolates)	ERIC-PCR type ID/ (no of isolates)		
		Human specific	Cattle specific	Shared between human and cattle
Bigwa	7(7)	46(1),47(1)	11(1),65(1)	8(1),53(1),56(1)
Boma	9(11)	7(1)	6(1),9(2),23(2), 27(1),61(1)	19(1),21(1),25(1)
Kichangani	4(4)		24(1)	12(1),19(1),25(1)
Kihonda	2(2)	46(1)		25(1)
Kihonda Magorofani	27(42)	2(1),31(1), 42(2),43(1),59(1),60(1)	3(1),5(1),16(1), 22(2),25(1),36(1), 51(5),54(3),55(2), 58(1),66(1)	8(2),12(1),21(1), 34(1),38)1),40(1), 48(2),49(3),53(1) 57(3)
Kingorwila	11(16)	35(1)	6(1),10(1),11(2), 20(3),26(1),30(1),	12(1),25(2),44(1), 56(2)
Mafisa	14(23)	1(1),62(1)	6(2),18(2),26(1), 27(2),29(1)	12(2),19(2),25(2), 34(2),38(1),49(3), 57(1),44(1)
Magadu	15(21)	43(1)	13(2),22(1),23(2), 37(1),39(1),63(1),	8(2),19(1),25(1), 38(1),45(1),49(2), 50(2),56(2)
Mazimbu	26(45)	4(1),33(1)	6(1),10(1),11(5), 14(1),15(2),17(1), 18(2),20(1),27(3), 28(1),30(2),32(1), 41(1),51(1),52(1), 64(1)	8(2),19(1),25(8), 38(1),40(2),45(2), 49(1),50(1),
Tungi	1(1)		27(1)	

In general, isolates belonging to the same genotype rarely shared the same antimicrobial resistance profile (data not shown). The level of antimicrobial resistance was most commonly higher among the isolates belonging to human-specific genotypes than to cattle specific or genotypes shared between human and cattle (data not shown). Based on this, further analysis of *E*. *coli* isolates representing the detected diversity in *E*. *coli* from cattle and cattle attendants in Tanzania, at the genome level, was performed. Five or six isolates from each of the three major ERIC-PCR groups (human specific, cattle specific and ERIC-PCR types shared between human and cattle) were selected for the genome sequencing in a way that each of them represented different ERIC-PCR cluster, geographical location and antimicrobial resistance profile (Table 4).

**Table 4:** Characteristics of 17 commensal *Escherichia coli* isolates from cattle and cattle attendants in Tanzania selected for whole genome sequencing.

Strain ID	Group	ERIC-PCR type	Source	Location	AR profile
BM233	Human	43	Human	Magadu	SXT/AMP
BM228	Human	46	Human	Bigwa	CN/TE/CIP/SXT/AMP
BM199	Human	33	Human	Mazimbu	TE
BM146	Human	35	Human	Kingorwila	Non-resistant
BM221	Human	60	Human	Kihonda Magorofani	Non-resistant
BM165	Human	62	Human	Mafisa	TE/SXT/AMP
BM224	Human&Cattle	25	Human	Kihonda Magorofani	TE/SXT/AMP
BM166	Human&Cattle	49	Human	Mafisa	Non-resistant
BM117	Human&Cattle	8	Cattle	Mazimbu	TE/AMP/AMC
BM116	Human&Cattle	12	Cattle	Kingorwila	Non-resistant
BM152	Human&Cattle	56	Human	Bigwa	SXT
BM447	Human&Cattle	19	Cattle	Boma	TE/C/AMP
BM33	Cattle	11	Cattle	Kingorwila	TE
BM449	Cattle	27	Cattle	Tungi	C/AMP
BM12	Cattle	20	Cattle	Mazimbu	Non-resistant
BM321	Cattle	51	Cattle	Kihonda Magorofani	TE/SXT/AMP
BM304	Cattle	37	Cattle	Magadu	Non-resistant

Two of the selected human associated *E*. *coli* strains represented ERIC-PCR genotypes 43 and 46. These types were each detected in two *E*. *coli* isolates from two different wards. The remaining isolates represented ERIC-PCR genotypes 33, 35, 60 and 62, each containing one *E*. *coli* isolate and originating from different wards. All selected human and cattle shared strains represented the most common genotypes found in this study, each demonstrated in isolates from up to four wards. Similarly, most cattle associated strains represented the most common genotypes 11, 27, 51, and 20, except 37, which was only detected in one ward (Table 4).

### *In silico* MLST, serotyping and phylogrouping

Draft genome assemblies representing 17 *E*. *coli* isolates were used to predict the ST type, serotype and phylogroup (Table 5). Each *E*. *coli* isolate was assigned to a distinct ST and serotype. There were five phylogroups (A, B1, C, D and E) identified among the isolates, and only one isolate was not assigned to any known phylogroup. The most common phylogroup was B1 representing six isolates from cattle, while two isolates from cattle and four from human isolate were placed in phylogroup A and C, respectively.

**Table 5:** *In silico* MLST, serotyping and phylogrouping of 17 genome sequenced commensal isolates of *E*. *coli* from cattle and cattle attendants in Tanzania.

Strain ID	SeroType Finder	MLST	Phylogroup*	
			2000	2013
BM233	O8:H9	ST-1139	A	A
BM228	O89:H10	ST-617	A	C
BM199	O8:H21	ST-3202	A	A
BM146	O1:H7	ST-59	D	D
BM221	O28ab:H9	ST-4741	A	C
BM165	O32:H36	ST-181	A	C
BM224	O45:H16	ST-69	D	E
BM166	O18:H55	ST-5303	A	A
BM117	O93:H16	new ST**	A	A
BM116	O150:H8	new ST**	B1	B1
BM152	O81:H27	ST-452	ND***	ND***
BM447	O88:H8	ST-297	B1	B1
BM33	H32	ST-5307	A	C
BM449	O117:H12	ST-101	B1	B1
BM12	O6:H21	ST-602	B1	B1
BM321	O128ab:H35	ST-1147	B1	B1
BM304	O29:H21	ST-58	B1	B1

*phylogroups identified based on Clermont *et al*. 2000 [23] and Clermont *et al*. 2013 [24].

**STs that are not yet defined due to the observation of novel alleles.

*** Phylogroup primer sequences not detected.

### Plasmid, antimicrobial resistance (AR) and virulence associated genes in *E*. *coli*

**Fig 1:**
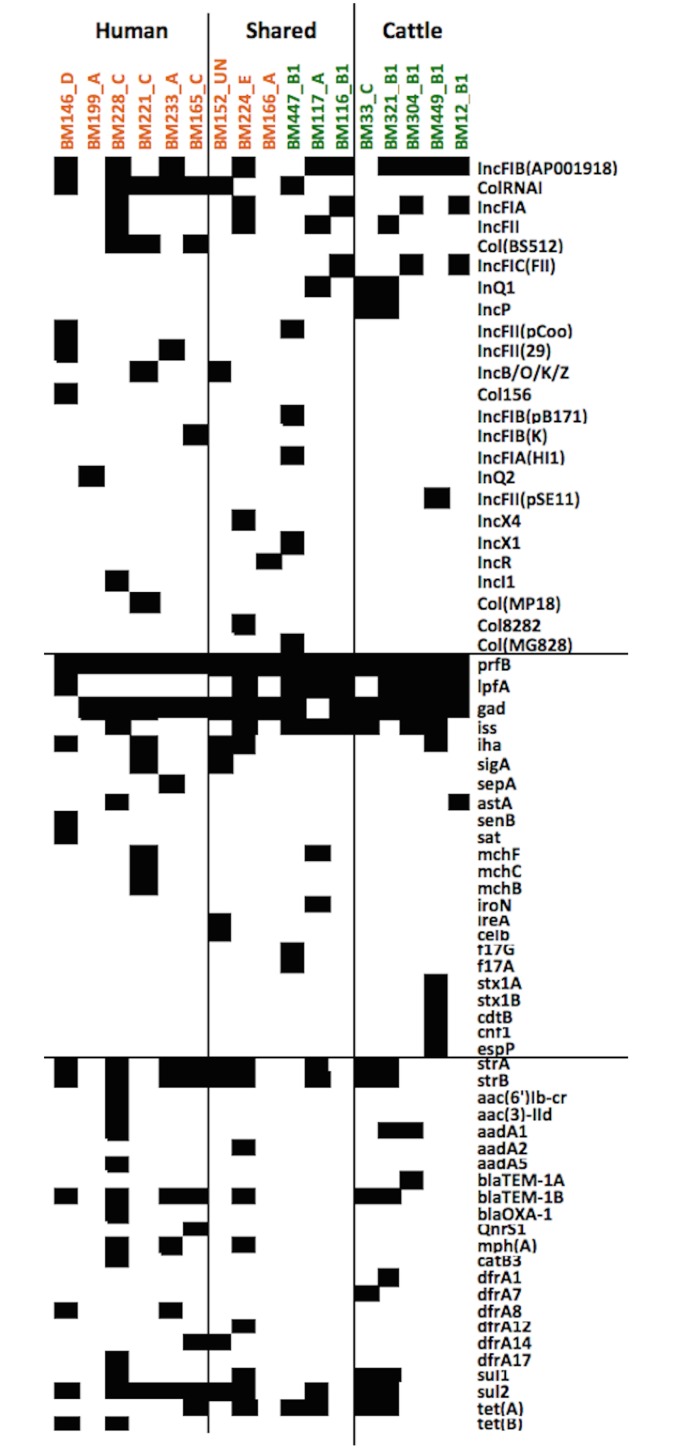
Virulence, resistance genes and plasmid replicons in 17 *E*. *coli* isolates. The presence of virulence, antibiotic resistance genes and plasmid replicons in 17 *Escherichia coli* genomes from cattle and cattle attendants in Tanzania in relation to their ERIC-PCR genotype, source of isolation and phylogenetic group. Black boxes show the presence and white boxes the absence of the relevant gene in each isolate. Isolates indicated with green were isolated from cattle, and isolates in orange—from human. Blocks indicate the host association of the strain based on ERIC-PCR.

Twenty-four different plasmid replicons were identified in the 17 genomes of *E*. *coli*, each isolate containing from one to six plasmid replicons, and there was no correlation between the presence of the specific replicon and the origin of the isolate (Fig 1).

*IncFIB*, *ColRNAI*, *IncFIA* and *IncFII* were the most common plasmid replicons detected in 10, 7, 5 and 4 isolates, respectively. The remaining replicons were found in ≤3 of the genomes. ResFinder identified up to 14 antibiotic resistance (AR) genes in single draft genomes of *E*. *coli* (Fig 1). On average from three to five genes per isolate were identified in cattle and human, respectively. The most abundant AR genes were *strA/strB*, *blaTEM-1B*, *sul2/sul1* and *tet(A)* encoding the resistance to streptomycin, beta-lactams, sulphonamides and tetracycline, respectively. The comparison of AR phenotype and *in silico* prediction results revealed agreement between phenotypic resistance to TE, CIP and C and the presence or absence of AR genes in the genome *(tet(A)*, *tet(B)*, *qnrS1*, *catB3*, *aac(6')lb-cr*,*aac(3)-lld)* with only two unexpected results in the form of presence of *tetB* in a tetracycline sensitive strain and *catB3* in a chloramphenicol sensitive strain. The presence of *qnrS1* on its own was not expected to cause ciprofloxacin resistance. However, more conflicts were found between the resistance to beta-lactams (AMP and AMC), Sulphamethoxazole:Trimethoprim (SXT) and gentamicin (CN) and the presence of AR gene in the genomes (Table 6).

**Table 6:** The correlation between the antibiotic resistance profile and *in silico* prediction of antimicrobial resistance genes in 17 sequenced isolates of commensal *E*. *coli* from cattle and cattle attendants in Tanzania.

Strain ID	SXT	TE	AMP	CIP	C	CN
BM233	+/ sul2/dfrA8*	-/-	+/blaTEM-1B	-/-	-/-	-/-
BM228	+/ sul1,sul2/dfrA17	+/tet(B)	+/blaTEM-1B, blaOXA-1	+/-	-/catB3	+/aac(6')lb-cr, aac(3)-lld,aadA1,aadA5
BM199	-/-/-	+/-	-/-	-/-	-/-	-/-
BM146	-/ sul2/dfrA8	-/tet(B)	-/blaTEM-1B	-/-	-/-	-/-
BM221	-/sul2/-	-/-	-/-	-/-	-/-	-/-
BM165	+/ sul2/dfrA14	+/tet(A)	+/blaTEM-1B	-/QnrS1	-/-	-/-
BM224	+/ sul1,sul2/dfrA12	+/tet(A)	+/blaTEM-1B	-/-	-/-	-/-aadA2
BM166	-/-/-	-/-	-/-	-/-	-/-	-/-
BM117	-/sul2/-	+/tet(A)	+/-	-/-	-/-	-/-
BM116	-/-/-	-/-	-/-	-/-	-/-	-/-
BM152	+/ sul2/dfrA14	-/-	-/-	-/-	-/-	-/-
BM447	-/-/-	+/tet(A)	+/-	-/-	+/-	-/-
BM33	-/ sul1,sul2/dfrA7	+/tet(A)	-/blaTEM-1B	-/-	-/-	-/-
BM449	-/-/-	-/-	+/-	-/-	+/-	-/-
BM12	-/-/-	-/-	-/-	-/-	-/-	-/-
BM321	+/ sul1,sul2/dfrA1	+/tet(A)	+/blaTEM-1B	-/-	-/-	-/-aadA1
BM304	-/-/-	-/-	-/blaTEM-1A	-/-	-/-	-/aadA1

*phenotype as shown in Table 4 indicated as + or—resistance/gene detected by ResFinder.

SXT—Sulphamethoxazole: Trimethoprim, Te—Tetracycline, AMP—Ampicillin, CIP—Ciprofloxacin, C—Chloramphenical, CN—Gentamycin

The analysis of *E*. *coli* genomes using VirulenceFinder tool revealed the presence of up to 10 virulence-associated genes in a single genome of *E*. *coli* (Fig 1). All the 17 isolates contained p-related fimbrial regulatory (*prfB)* gene. The second most frequent gene was *gad*, which was absent in two genomes, one *E*. *coli* from human and the other from cattle. The third and fourth most common genes *lpfA* and *iss* were found to be predominant among the cattle isolates (present in 7/8 isolates and 6/8 isolates, respectively), and were present only in two human isolates. Four human and one cattle isolate had the *iha* gene encoding adhesion, which is an important virulence factor of uropathogenic *E*. *coli*; however only one *E*. *coli* isolate from cattle carried a copy of *E*. *coli* toxins (*stx1A*, *stx1B*, *cdtB* and *cnf1*). One human and one cattle isolate carried *astA* gene, encoding heat-stable enterotoxin 1 (EAST1) of *E*. *coli*. All 17 *E*. *coli* isolates carried the gene encoding the *Shigella* enterotoxin ShET-2.

### Evolutionary relationship analysis using conserved Signature Indels (CSItree)

**Fig 2:**
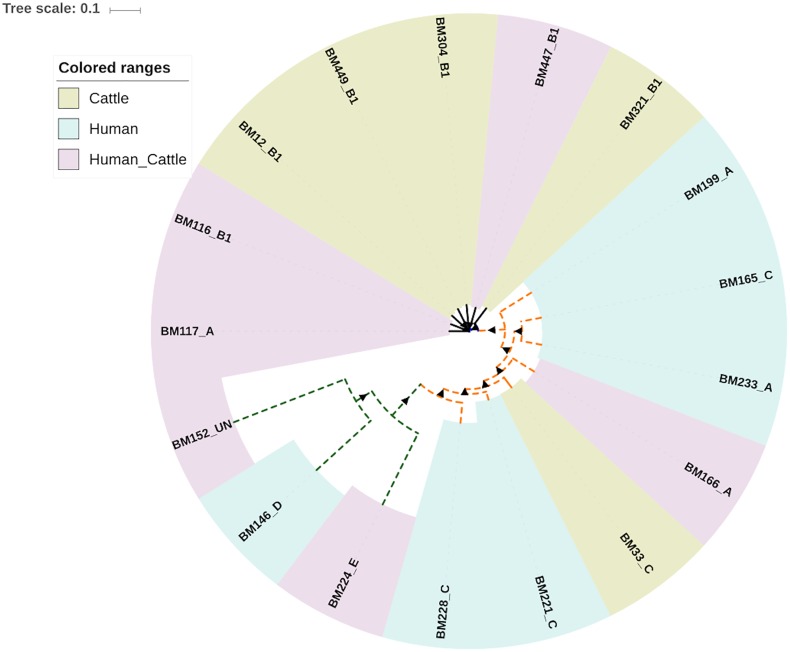
Evolutionary relationship of 17 commensal *E*. *coli* isolates sequenced in this study. Evolutionary relationship between 17 sequenced strains of *Escherchia coli* from cattle and cattle attendant in Tanzania was inferred using CSI phylogeny. Three clusters I, II and III are indicated in green, blue and orange lines respectively. Isolates obtained from human are indicated with dashed lines, and from cattle—with solid lines. The coloured ranges represent either host specific or shared ERIC-PCR types. Triangles show bootstrap support in a range from 87 to 100. An image was created using iTOL [29].

Two different methods were deployed to reconstruct the evolutionary relationship between the 17 commensal *E*. *coli* isolates sequenced in this study; the 7 gene MLST-based approach and the core genome SNPs-based approach using CSI phylogeny. The genomes were grouped into three major clusters according to both methods. Cluster I included three human isolates representing phylogroup E and D, cluster II included seven cattle isolates assigned to phylogroup B1 (except one isolate assigned to phylogroup A), and cluster III, which was composed of six human isolates, and one cattle isolate. Strains within cluster III were assigned to either phylogroup A or C (Fig 2).

### Host and lineage specific gene detection

The pan-genome of the 17 strains was calculated to consist of 9310 genes. The pan-genome was growing by 0.9-fold with the addition of the new strain to the analysis, and at the end it was still increasing (S1 Fig). The comparison of genomes, grouped based on the ERIC-PCR clustering (human, cattle/human, cattle) or based on the source of isolation (human, cattle), did not reveal any genome content differences between the different sample sets. The highest proportions of specific genes were identified when strains belonging to clusters I, II and III, as defined by MLST and CSI phylogeny were compared (S2 Fig).

## Discussion

*E*. *coli* species is abundant and exhibit high diversity in the GIT of many different hosts [30, 31]. Several authors have reported on the ability of commensal *E*. *coli* to transform into pathogenic types, and to exchange antimicrobial resistance genes, especially in hosts with lowered immunity [14, 30]. This study aimed at characterizing genomes of *E*. *coli* isolated from cattle and healthy humans in contact with these animals to determine the apparent host association of commensal *E*. *coli*, their pathogenic potential and the degree to which they share genomic contents.

In this study typing by ERIC-PCR method was applied to characterize *E*. *coli* isolates representing cattle and cattle attendants from different farms in Tanzania. Overall 66 ERIC-PCR clusters, of which 16 clusters were shared between human and cattle, were identified after this analysis. It is important to note that *E*. *coli* isolates collected in this study do not represent the full *E*. *coli* diversity present in the gastrointestinal tract of apparently healthy human and cattle at the time of sampling, since only one colony was obtained per animal/humans. On average, humans have been found to carry up to 3.5 genotypes of *E*, *coli* and cattle to carry 3.4 genotypes per individual, and a coverage of the full diversity would have required collecting >5 different colonies per sample [32, 33]. Despite this limitation, the detection of the same *E*. *coli* genotypes in humans and cattle in the current study demonstrate that some *E*. *coli* populations may colonize the intestine of different hosts in the same geographic area, and thus may potentially be the source of exchange of genetic material between such hosts.

Before genotyping, the *E*. *coli* isolates were screened for resistance to ten antimicrobials. This analysis demonstrated that human isolates were more resistant compare cattle isolates, and in support to previous studies [1, 32], the resistance profile was ERIC-PCR genotype independent. The more detailed analysis showed that there was higher antimicrobial resistance among human isolates particularly belonging to human specific genotypes. This finding is in contrary to previous findings [12], where it was shown that antimicrobial resistance in poultry isolates correlated with antimicrobial resistance in strains from animal attendants. While the tendency is to assume that farm animals are subjected to a higher antibiotic pressure than humans, our results indicate that this may not be the case under extensive, livestock production as practiced in developing countries. Under such circumstances, humans may constitute a higher risk to cattle than vice versa with respect to transfer of antimicrobial resistant bacteria. In contrast to this, smaller resistomes in human than in animal isolates have been previously detected in the study of Escobar-Paramo [34]; this study also suggested that increase of AR genes in human isolates may be the result of poor hygiene, exposure to various antibiotics or constant contact with animal host. In our case, the results indicated that human specific genotypes might have gained higher antimicrobial resistance, probably due to more frequent antimicrobial treatment of humans than cattle in Tanzania. The concern is, that the genome content of these genotypes may be transferred to *E*. *coli* capable of colonizing both hosts, and thus increase virulence and antimicrobial resistance of previously less harmful isolates. To investigate if there is such possibility, six genomes representing *E*. *coli* genotypes that were only detected among humans in the current study were compared with five and six genomes representing cattle specific genotypes and genotypes shared between human and cattle, respectively.

*E*. *coli* subjected to whole genomes sequencing were selected to have low ERIC-PCR profile similarity and therefore each was assigned to unique ST- and serotype using *in silico* analysis tools. Some sero-types such as O8 and O6, representing two human isolates and one cattle isolate, are commonly associated with ETEC from outbreaks as well as from water in the environmental [35, 36]. The most important virulence factors of ETEC are various adhesions and enterotoxins, however none of the isolates carried ETEC associated enterotoxins *sta1* or *sta2*. Among the identified STs, ST-617 representing human isolate and phylogroup A, has recently been found to be associated with *E*. *coli* producing CTX-M-15 in hospitalized patients Mauritania [37]. One human-specific strain (BM146) was assigned to ST-59, of O1:H7 and phylogroup D, which was previously found to be a prevalent clone among the human ExPEC and suggested to be human specific pathotype [38]. However only human strains and poultry APEC isolates were compared in that study. Mora et al. (2011) [39] also reported this *E*. *coli* clone to be one of the most prevalent ExPEC clones producing CTX-M-14 in Spain and harbouring more than eight virulence factors. BM224 representing the most frequent ERIC-PCR genotype 25 shared between human and cattle was assigned to ST-69 and phylogroup D. This *E*. *coli* clone was frequently detected among human UTI *E*. *coli* isolates clustering closely with cow isolate in US [40]. Noteworthy, ST-69 has enhanced ability to colonize, persist and adapt to different hosts and contributed largely to the dissemination of β-lactam resistance determinants (mainly extended-spectrum β-lactamases and/or carbapenemases) in different countries [41]. Another interesting clone observed, ST-101, representing cattle specific ERIC-PCR genotype and phylogroup B1, is globally spread and strongly associated with human ExPEC and production of CTX-M-14 and NDM-1 [42]. Other STs, such as ST-602 and ST-58 have been described to be associated with carriage of CTX-M-1 [43, 37]. In the current study, *E*. *coli* isolates BM12 and BM304 belonging to phylogroup B1 from cattle, were of these types. Overall, ST and serotype distribution among *E*. *coli* isolates examined in this study indicates that both human and cattle *E*. *coli* in Tanzania exhibit pathogenic potential to humans and the ability to disseminate antimicrobial resistance genes within and between different hosts.

Differences in phylogroups distribution were found among the *E*. *coli* genomes representing the two different hosts. The majority of cattle isolates where assigned to group B1, whereas human isolates where assigned to C, A, D and E groups. A, C and B1 were previously found to be most prevalent and associated with commensal strains of *E*. *coli* in both human and animal hosts [23, 24, 44–46]. However, Escobar-Paramo et al. (2006) [34] demonstrated that the predominant commensal *E*. *coli* phylogroups in human are A/B2 and in non-human mammals are A/B1, which is in agreement with our findings.

*In silico* detection of *E*. *coli* virulence determinants demonstrated their random distribution between the *E*. *coli* genotypes representing cattle and humans associated genotypes. Virulence factors *stx1A*, *stx1B and astA* associated with enterohemorrhagic (EHEC) and enteroaggregative (EAEC) pathotypes of *E*. *coli* were identified in several *E*. *coli* isolates. Toxin-encoding genes were detected in only few isolates from cattle. This is contrary to our expectations, as frequent carriage was previously reported in Iran, Japan and China [47–49]. *Shigella* enterotoxin *ShET-2* was present in all *E*. *coli* genomes. This gene has been found to be highly prevalent in *Shigella* isolates [50, 51] as well as among EIEC, EAEC, ETEC-ST, and *E*. *coli* isolates not associated to diarrhea [52] and *E*. *coli* associated with bacteremia, however the role of this toxin in bacteremia as well as in other infections caused by *E*. *coli* is not yet revealed [53]. In contrast to the latter study, where *ShET-2* was predominant in phylogroup B1, we found it to be present in *E*. *coli* isolates representing different phylogroups. Manual search of genes *ihaA*, *eae*, *est*, *stx*, *elt*, *aggR*, *aspU* and CVD432 using primers published by Toma et al. (2003) [54] allowed identification of EAEC associated genes *aggR*, *aspU* and CVD432 in one human isolate BM233. This isolate was assigned to serotype O8:H9, which was previously strongly associated with ETEC [35]. In total five genes (*prfB*, *lpfA*, *iha*, *f17G* and *f17A*) encoding proteins playing role in the adhesion of *E*. *coli* were found in the genomes examined in this study. Two genes encoding the adhesion activator *prfA* and long polar fimbriae *lpfA* were previously found to be prevalent in clinical and commensal *E*. *coli* isolated from human and bovine hosts [55]. In agreement with the previous report [44, 56] we found *lpfA* to be predominant among the isolates assigned to phylogroup B1. Blum et al. (2013) [44] demonstrated that *lpfA*, together with *iss* (responsible for increased serum survival) and *astA* were the most prevalent virulence factors in *E*. *coli* associated with mastitis. In agreement to this, we found *lpfA* and *iss* to be more abundant in cattle than in human isolates, which supports that they may be important for colonization of cattle. The adhesins *iha*, *f17G* and *f17A*, on the other hand were less prevalent in *E*. *coli* genomes in the current study. *iha*, found in several human and one cattle isolate, was shown to play role in urinary tract infections in human and pigs [57, 58] and *f17G* and *f17A* to be present in *E*. *coli* isolates pathogenic to ruminant hosts specifically [59]. Collectively, virulence factor analysis indicate that *E*. *coli* populations in healthy cattle and humans carried various virulence factors associated with intra- and extra-intestinal *E*. *coli* pathotypes, and all of them contained enterotoxin ShET -2. Importantly, most of the identified virulence factors are plasmid associated and thus may be transferred horizontally between the strains. Commensal *E*. *coli* isolated from healthy cattle and human may act as a source of virulence factors to other *E*. *coli* strains present in the gut, and conversely, such commensal strains may acquire several virulence factors from other strains, and may transform into a pathogen [60, 61].

Further analysis of the *E*. *coli* genomes revealed that human isolates carried more antimicrobial resistance genes compare to cattle. Since initially the selected human and animal isolates showed similar AR profile (resistance to 2–3 antibiotics/per isolate), this was not the reason for the difference detected. One reason of this difference is that a number of human *E*. *coli* genomes additionally carried genes encoding resistance to streptomycin (*strA/strB*) and macrolides (*mphA*), which was not tested phenotypically. In addition, in some cases the genes (*aadA2*, *blaTEM1B*, *dfrA8*, *sul2*, *tet(B)*) encoding resistance to the tested antimicrobials were present, however phenotypic resistance was not found. It may be explained by the fact that some genes, even though present in the genome, may not be expressed due to mutations or disruptions or would be expressed only under certain conditions. Additionally, certain chromosomally encoded antimicrobial resistance genes are not reported by ResFinder [20].

In agreement to a previous report [62], the *strA* and *strB* genes were often found together with sulphonamide and tetracycline resistance genes, *sul1/sul2* and *tet(A)/tet(B)*, respectively. Moreover, in five isolates *strA*, *strB* and *sul1/sul2* and in some cases *tet(A)* and trimethoprim endcoding genes *dfrA* where found to be on the same contigs associated to plasmids of *IncQ1* and *ColRNAI* type. *IncQ1* was associated with *E*. *coli* resistance to streptomycin and sulphonamides previously [20]. In some isolates *tet(A)* was located on the same contigs as plasmids of *IncP* and *IncFIB* type, and *IncFIB* was the most common plasmid detected in *E*. *coli* genomes in the current study. Plasmids of this type are highly prevalent in fecal flora of humans and animals and may carry both virulence and antibiotic resistance genes [63]. In addition to *IncFIB* type plasmids, plasmids of *IncFIA*, *IncFIC* and *IncFII* type associated with resistance to beta-lactams were also detected. All isolates containing these plasmids also carried genes *bla*_*TEM-1A*_, *bla*_*TEM-1B*_ and *bla*_*OXA-1*_ encoding beta-lactamases of *E*.*coli*. Both *bla*_*TEM-1A*_ and *bla*_*TEM-1B*_ molecular variants were found to be common among ampicillin susceptible and ampicillin resistant isolates of human and animal origin [64]. Similarly, 3/8 isolates carrying one of these genes did not show resistance to beta-lactams phenotypically. The ampicillin susceptibility of isolates carrying *bla*_TEM_ genes may be explained by the poor expression of functional enzyme due to mutations in the promoter region or due to the production of inactive enzyme as it was shown in case of *Haemophilus influenzae* [65].

Among the ampicillin resistant *E*. *coli*, we detected three isolates with no ampicillin resistance genes identified by the ResFinder. It is likely that these isolates carry chromosomal ampicillin resistance gene *ampC* [66], which is not reported by the ResFinder. Overall, most of sequenced *E*. *coli* isolates from healthy human and cattle showing resistance to single or multiple antibiotics carried antibiotic resistance associated genes and resistance associated plasmids. Such *E*. *coli* isolates may play as a source of antimicrobial resistance genes for other *E*. *coli* strains or strains of closely related genera.

Finally, the analysis of genome content between *E*. *coli* isolates from different hosts did not reveal the presence of human or cattle host specific genes. This may indicate that in this case strains are not host specific and their association with a particular host in the current study was an artifact of the sampling strategy, or host specificity may not be detectable by the presence or absence of one particular gene. Contrary to this, a number of cluster or phylogroup specific genes were identified, supporting previous hypothesis of the presence of coevolution between the chromosomal background and the flexible gene pool in the isolates belonging to different phylogenetic groups [67]. This shows that lineage development is both an ancestral feature and that is not coincidental. In other words, certain phylogroups have evolved over long period of time, whereas specific strain properties were obtained through horizontal gene transfer. These findings are in support to other studies investigating the evolution of *E*. *coli* [34].

## Conclusions

In conclusion, highly diverse population of commensal *E*. *coli* was found in cattle and cattle attendants in Tanzania. Despite this, a number of ERIC-PCR types shared between both hosts in same location were detected, suggesting that humans in close contact with cattle share commensal *E*. *coli* types with these animals. Virulence factor profiles were host independent and higher frequency of phenotypic resistance to antimicrobials and carriage of AR genes was detected in cattle attendants than in cattle. This indicates that human may be exposed more to antimicrobials and thus may acquire higher resistance. The predominance of the same types of plasmids generally associated with carriage of virulence and antimicrobial genes in both hosts suggests frequent gene exchange. Further studies are recommended to investigate the source of pathogenic factors in commensal *E*. *coli*, whether be it from cattle to human or vice versa.

## Supporting Information

S1 Fig The pan- and core-genome of 17 commensal isolates of *E*. *coli* from cattle and cattle attendants in Tanzania. A: on the left, the intersect pan-genome containing 9310 orthologs was calculated using COG and OMCL algorithms. On the right, Tettelin graph showing an increase of pan-genome with the addition of new isolate to the analysis. B: on the left, the intersect core-genome containing 3016 orthologs was calculated using COG, OMCL and BDBH algorithms. On the right, Tettelin graph showing the decrease of the core-genome with the addition of new isolate to the analysis.
(TIF)

S2 Fig *E*. *coli* lineage specific genes in 17 commensal isolates of *E*. *coli* from cattle and cattle attendants in Tanzania. The matrix shows the number of genes present in 90% of the isolates assigned to phylogenetic clusters indicated on the left column and absent in 90% of the isolates assigned to phylogenetetic clusters indicated on the upper row.
(PDF)

S1 Table Detailed statistics of genomes from 17 commensal *Escherichia coli* strains sequenced in this study. (DOCX)
