# Supplementary figures and images for: Characterisation of Commensal Escherichia coli Isolated from Apparently Healthy Cattle and Their Attendants in Tanzania
Source: PLoS One. 2016 Dec 15;11(12):e0168160. doi: 10.1371/journal.pone.0168160 (PMC5158034; doi:10.1371/journal.pone.0168160)

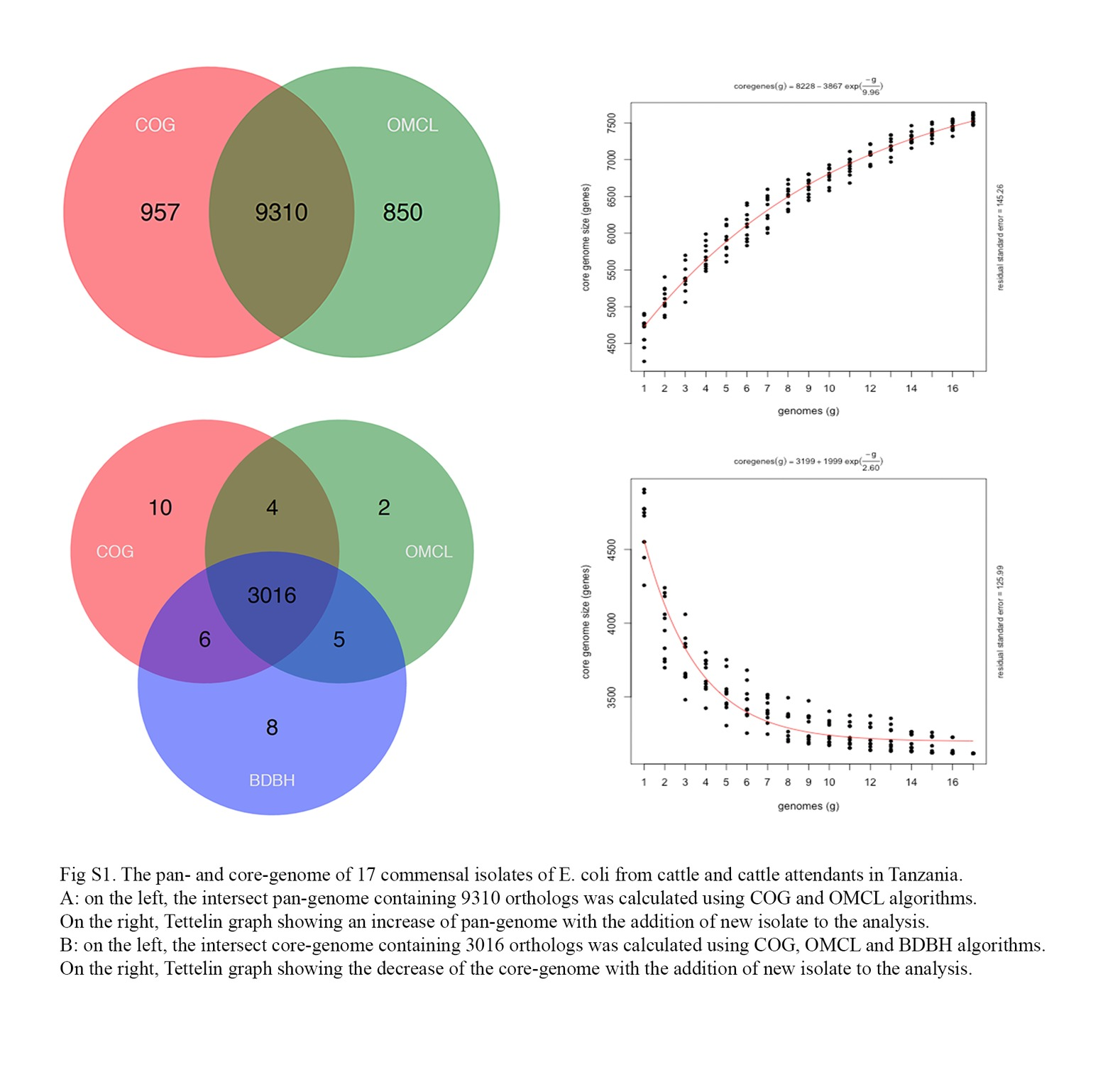

Supplement: S1 Fig — A: on the left, the intersect pan-genome containing 9310 orthologs was calculated using COG and OMCL algorithms. On the right, Tettelin graph showing an increase of pan-genome with the addition of new isolate to the analysis. B: on the left, the intersect core-genome containing 3016 orthologs was calculated using COG, OMCL and BDBH algorithms. On the right, Tettelin graph showing the decrease of the core-genome with the addition of new isolate to the analysis. (TIF) [file pone.0168160.s001.tif]
