# Supplementary material for: Characterisation of Commensal Escherichia coli Isolated from Apparently Healthy Cattle and Their Attendants in Tanzania
Source: PLoS One. 2016 Dec 15;11(12):e0168160. doi: 10.1371/journal.pone.0168160 (PMC5158034; doi:10.1371/journal.pone.0168160)
Supplement: S2 Fig — The matrix shows the number of genes present in 90% of the isolates assigned to phylogenetic clusters indicated on the left column and absent in 90% of the isolates assigned to phylogenetetic clusters indicated on the upper row. (PDF) [file pone.0168160.s002.pdf]

	cI	cII	cIII	cIcII	cIcIII	cIIcIII
**cI**		72	48			**47**
**cII**	100		30		**18**	
**cIII**	54	30		**17**		
**cIcII**			**19**			
**cIcIII**		**21**				
**cIIcIII**	**55**					

Fig S2. E. coli lineage specific genes in 17 commensal isolates of E. coli from cattle and cattle attendants in Tanzania. . The matrix shows the number of genes present in 90% of the isolates assigned to phylogenetic clusters indicated on the left column and absent in 90% of the isolates assigned to phylogenetetic clusters indicated on the upper row.
