# Supplementary material for: Characterisation of Commensal Escherichia coli Isolated from Apparently Healthy Cattle and Their Attendants in Tanzania
Source: PLoS One. 2016 Dec 15;11(12):e0168160. doi: 10.1371/journal.pone.0168160 (PMC5158034; doi:10.1371/journal.pone.0168160)
Supplement: S1 Table — (DOCX) [file pone.0168160.s003.docx]

**S1 Table.** **Detailed statistics of genomes from 17 *Escherichia coli* strains sequenced in this study**

| **Strain**  **ID** | **No. of reads**  **after quality trimmming** | **Average**  **read length** | **Genome**  **size, Mb** | **Coverage** | **No of**  **contigs** | **G+C %** | **Genes** | **CDS** | **Pseudo**  **genes** | **tRNAs** | **Accession**  **number** |
| --- | --- | --- | --- | --- | --- | --- | --- | --- | --- | --- | --- |
| BM233 | 2421006 | 188 | 4,9 | 93,43 | 131 | 50,61 | 4932 | 4687 | 135 | 87 | LIVH00000000 |
| BM228 | 2138096 | 197 | 5,1 | 83,20 | 169 | 50,73 | 5141 | 4921 | 105 | 86 | LIVG00000000 |
| BM199 | 2161088 | 191 | 4,6 | 89,29 | 157 | 50,91 | 4645 | 4397 | 142 | 83 | LIVD00000000 |
| BM146 | 2419126 | 198 | 5,2 | 92,92 | 171 | 50,60 | 5262 | 5029 | 135 | 82 | LIUZ00000000 |
| BM221 | 2161014 | 191 | 5,2 | 79,98 | 269 | 50,70 | 5353 | 5092 | 129 | 98 | LIVE00000000 |
| BM165 | 2192110 | 191 | 4,7 | 89,09 | 123 | 50,81 | 4705 | 4480 | 116 | 85 | LIVB00000000 |
| BM224 | 2220880 | 193 | 5,3 | 81,22 | 213 | 50,62 | 5417 | 5197 | 103 | 90 | LIVF00000000 |
| BM166 | 2408660 | 195 | 4,8 | 98,42 | 181 | 51,01 | 4802 | 4574 | 117 | 88 | LIVC00000000 |
| BM117 | 2131368 | 198 | 4,9 | 85,97 | 105 | 50,67 | 4951 | 4756 | 79 | 84 | LIUY00000000 |
| BM116 | 1965946 | 200 | 4,8 | 82,02 | 95 | 50,60 | 4810 | 4634 | 72 | 77 | LIUX00000000 |
| BM152 | 2071280 | 198 | 5,0 | 82,28 | 239 | 50,59 | 5151 | 4879 | 176 | 78 | LIVA00000000 |
| BM447 | 2180226 | 196 | 5,0 | 85,38 | 117 | 50,53 | 5078 | 4901 | 75 | 75 | LIVK00000000 |
| BM33 | 2164134 | 198 | 4,7 | 90,22 | 88 | 50,79 | 4706 | 4531 | 70 | 81 | LIUW00000000 |
| BM449 | 2505078 | 198 | 5,0 | 99,33 | 149 | 50,74 | 5055 | 4834 | 110 | 79 | LIVL00000000 |
| BM12 | 1858848 | 196 | 5,3 | 68,96 | 206 | 50,55 | 5414 | 5181 | 118 | 86 | LIQA00000000 |
| BM321 | 2091356 | 198 | 4,8 | 86,54 | 90 | 50,79 | 4817 | 4644 | 72 | 82 | LIVJ00000000 |
| BM304 | 2297638 | 196 | 5,5 | 82,16 | 250 | 50,53 | 5673 | 5426 | 133 | 80 | LIVI00000000 |
